# Spatial Memory in Spontaneously Hypertensive Rats (SHR)

**DOI:** 10.1371/journal.pone.0074660

**Published:** 2013-08-29

**Authors:** Thomas-A. Sontag, Anselm B. M. Fuermaier, Joachim Hauser, Ivo Kaunzinger, Oliver Tucha, Klaus W. Lange

**Affiliations:** 1 Department of Experimental Psychology, University of Regensburg, Regensburg, Germany; 2 Department of Clinical and Developmental Neuropsychology, University of Groningen, Groningen, The Netherlands; Western University of Health Sciences, United States of America

## Abstract

The spontaneously hypertensive rat (SHR) is an established animal model of ADHD. It has been suggested that ADHD symptoms arise from deficits in executive functions such as working memory, attentional control and decision making. Both ADHD patients and SHRs show deficits in spatial working memory. However, the data on spatial working memory deficits in SHRs are not consistent. It has been suggested that the reported cognitive deficits of SHRs may be related to the SHRs’ locomotor activity. We have used a holeboard (COGITAT) to study both cognition and activity in order to evaluate the influence of the activity on the cognitive performance of SHRs. In comparison to Wistar-Kyoto (WKY) rats, SHRs did not have any impairment in spatial working memory and reference memory. When the rats’ locomotor activity was taken into account, the SHRs’ working memory and reference memory were significantly better than in WKY rats. The locomotor activity appears to be a confounding factor in spatial memory tasks and should therefore be controlled for in future studies. In the SHR model of ADHD, we were unable to demonstrate an impairment of working memory which has been reported in patients with ADHD.

## Introduction

Several animal models of attention deficit hyperactivity disorder (ADHD) have been proposed [1,2]. The spontaneously hypertensive rat (SHR) was developed by inbreeding rats of the Wistar-Kyoto (WKY) strain and is one of the best-studied animal models of ADHD [1]. In comparison to WKY rats, SHRs show various behavioural alterations characteristic of ADHD, including hyperactivity, impulsivity, poor sustained attention and impaired ability to withhold responses [3–9]. In addition, there is increasing evidence that SHRs also show impairments in learning and memory [7,10–17]. Deficits in learning and memory have also been reported in patients with ADHD [18]. Furthermore, it has been suggested that ADHD symptoms arise from deficits in executive functions such as working memory, attentional control and decision making [19]. For example, patients with ADHD have been reported to show deficits in verbal and spatial working memory [20,21]. Given the hypothesis that the SHR is an animal model of ADHD, memory deficits of SHRs such as those reported above might reflect ADHD-like deficits. Furthermore, if executive dysfunctioning is important in ADHD, one might expect to find deficits of working memory in SHRs.

The published studies on working memory in SHRs reveal inconsistent findings. For example, using the radial arm maze, Mook et al. [22] showed better working memory performance of SHRs compared to WKY rats, while Wyss et al. [16] showed, in comparison to Sprague-Dawley rats, a worse performance of SHRs aged 12 months and a better performance at the age of 3 months. Other studies reported more working memory errors of SHRs compared to WKY rats [12,23]. Using a delayed non-matching-to-position task, De Bruin et al. [24] reported a reduced accuracy in SHRs compared to WKY rats. In the delayed matching-to-place version (DMP) of a water maze, one study reported longer latencies for finding the hidden platform in SHRs compared to Sprague-Dawley rats and shorter latencies than in WKY rats [15]. Other findings have suggested that SHRs learn the DMP task better since their swim distances are shorter in the recall trials [25]. Given the fact that the SHRs show hyperactive behaviour [8,26,27] it cannot be ruled out that this behaviour has an influence on the cognitive performance of SHRs [28].

In the present experiment, a holeboard with a fixed pattern of hidden food pellets was used to assess the cognitive performance of SHRs. This system was chosen because spatial working memory error and spatial reference memory error can be assessed simultaneously and can be expressed in relation to the general activity of the animals tested. This is important with respect to the hyperactivity of SHRs. We hypothesised that the hyperactivity of the SHRs is a confounding factor when assessing spatial memory errors.

## Materials and Methods

All experiments were performed in accordance with the national laws (German law on Protection of Animals) and the principles of laboratory animal care (NIH publication No. 86-23, revised 1985). Before the start of the experiment the protocol of this study was submitted to the animal welfare officer of the University of Regensburg. In accordance to the national law of animal protection the animal welfare officer decided that this study did not require an approval by the competent authority because this study do not include any significant suffering or pain for the animals. Based on this decision and according to the national law there was no need to inform the competent authority about this study. Since the animals did not experienced any pain it was no necessary to take any measure to ameliorate it. For the animals welfare the weight and the health of the animals were controlled each day. At the end of the experiment the animals were sacrificed using CO2.

### Animals and feeding procedure

Fifteen male SHRs and 15 male WKY rats were used in this experiment. All animals were aged about three months at the beginning of the experiment. The rats were delivered by Charles River (Sulzbach, Germany). The animals were kept on a 12: 12 h light-dark cycle (room temperature 21 ^0^C, humidity 55%) and water was provided ad libitum. Since the learning on the holeboard is based on food reward, the rats were put on mild food restriction during the week prior to testing with the COGITAT System and throughout the subsequent test period. See also 29–31. The rats’ body weight was carefully controlled and a weight reduction of more than 10-15% compared to free fed animals was avoided in order to prevent stress [32,33] and subsequent changes in the dopaminergic system [34]. The animals received their daily food ration (15-20 g/rat/day) after the test sessions.

All experiments were performed in accordance with the national laws (German law on Protection of Animals) and the principles of laboratory animal care (NIH publication No. 86-23, revised 1985).

### COGITAT Holeboard (Figure 1)

**Figure 1:**
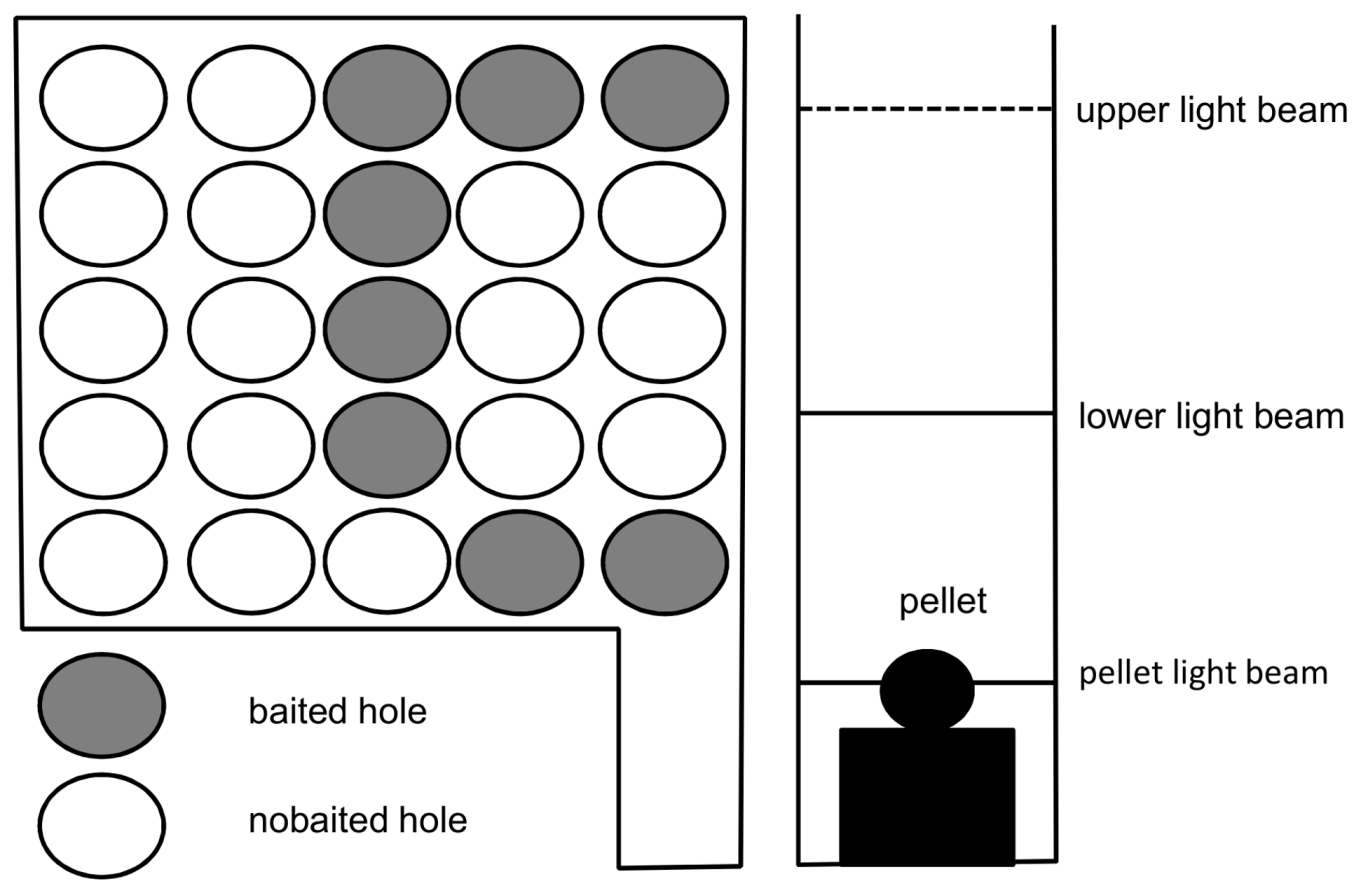
Schematic drawing of the COGITAT holeboard.

The performance of the rats was tested with the COGITAT Holeboard System (Cogitron GmbH, Göttingen, Germany. This system consists of a board (size 825 x 825 mm) with 25 holes (diameter 60 mm). Each hole of the board is closed at its lower end by an adjustable feeding plate with a depression for a food pellet. Feeding plate and food pellets are of the same color. The ground below the feeding plate is covered with the same pellets as those used in the cylindrical tubes, in order to prevent the animals from finding the pattern of the pellet distribution by using olfactory stimuli. Each hole is fitted with infrared light beams at different levels of the hole to measure the activity at the holes. Furthermore, there is an infrared beam at the feeding plate measuring the collection of a food pellet. For details see 35. In this study, eight of the 25 holes were baited. A search trial was automatically finished when a rat had found all the hidden pellets or after a fixed period of time (60 s). In each single trial, the following parameters were measured: (1) working memory error (i.e. the percentage of visits to previously baited and emptied holes in relation to the total number of holes visited in a single trial); (2) reference memory error (i.e. the percentage of visits to non-baited holes in relation to the total number of holes visited in a single trail). In addition, all trials were recorded with a video system. These data were digitalized and analyzed using the video tracking system ETHOVISION 3.0 (Noldus, Wageningen, The Netherlands). The route covered in each trial was measured with the COGITAT system in order to assess the rats’ activity.

### Training and test procedure

Prior to the learning of the pattern, the rats were habituated to the holeboard in order to reduce stress during the behavioural testing. The habituation lasted three minutes once daily for 10 days. Eight of the 25 holes were baited at random. After the habituation all rats were trained on a fixed pattern (8 holes were baited) with a maximal trial length of one minute for 35 days. The rats had one trial per day. The animals were trained on the board in a randomized order to avoid systematic circadian effects.

### Statistics

The performance of each rat was analyzed using the average of the last 10 trials in order to reduce the potential influence of different learning abilities of the rats. Results are expressed as means ± standard errors (M ± SE). Possible differences between SHRs and WKY rats regarding the route covered were analyzed with Student’s t-test for independent samples. The differences between the groups regarding working memory and reference memory errors were evaluated using analysis of variance (ANOVA) and analysis of variance with the covariate route covered (ANCOVA). The analyses were performed with the Statistical Package for Social Sciences 19.0 (SPSS) for Windows. An alpha level of 0.05 was applied.

## Results

**Figure 2:**
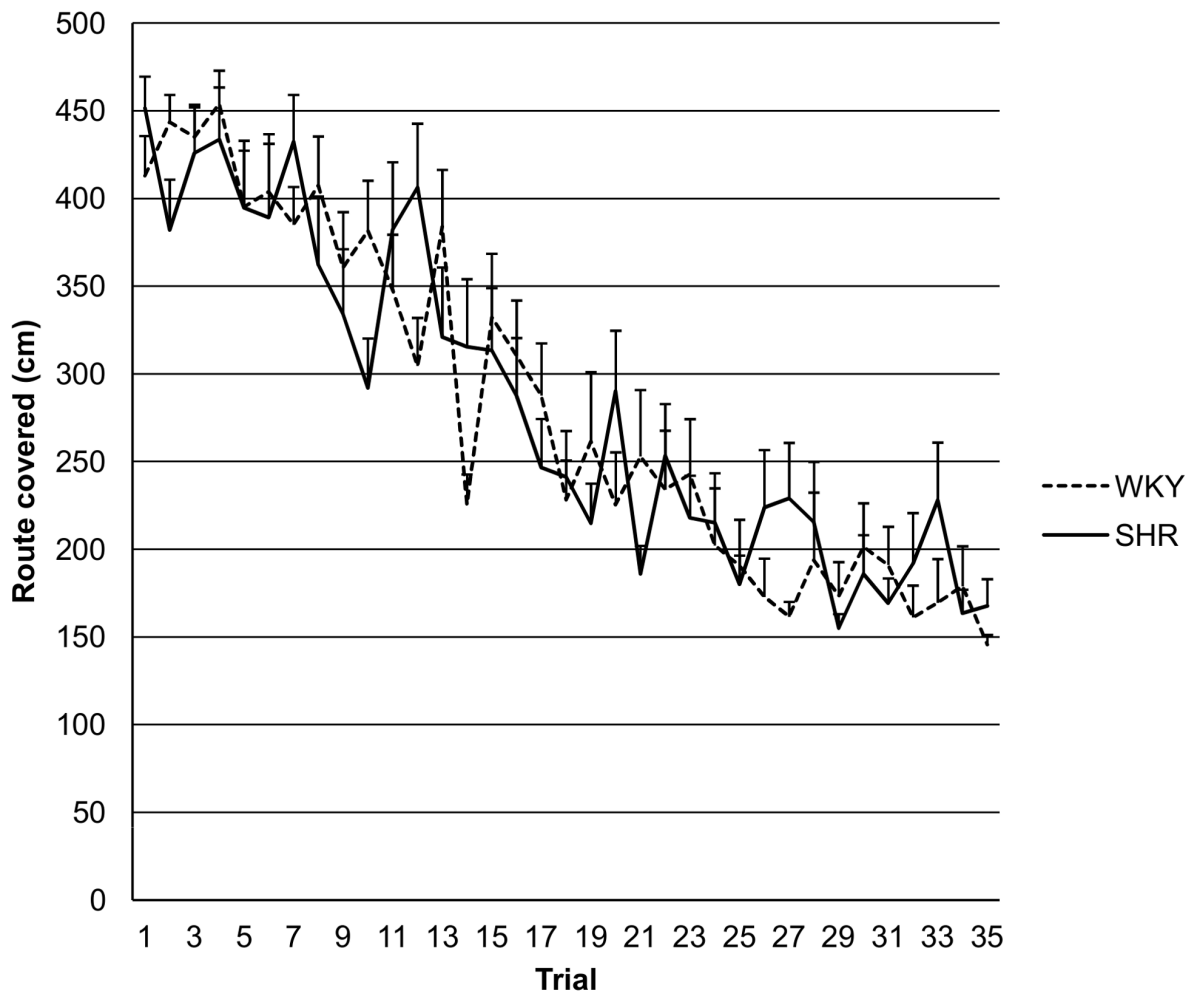
The route covered for WKY rats and SHRs. Values are means ± standard errors for each trial.

**Figure 3:**
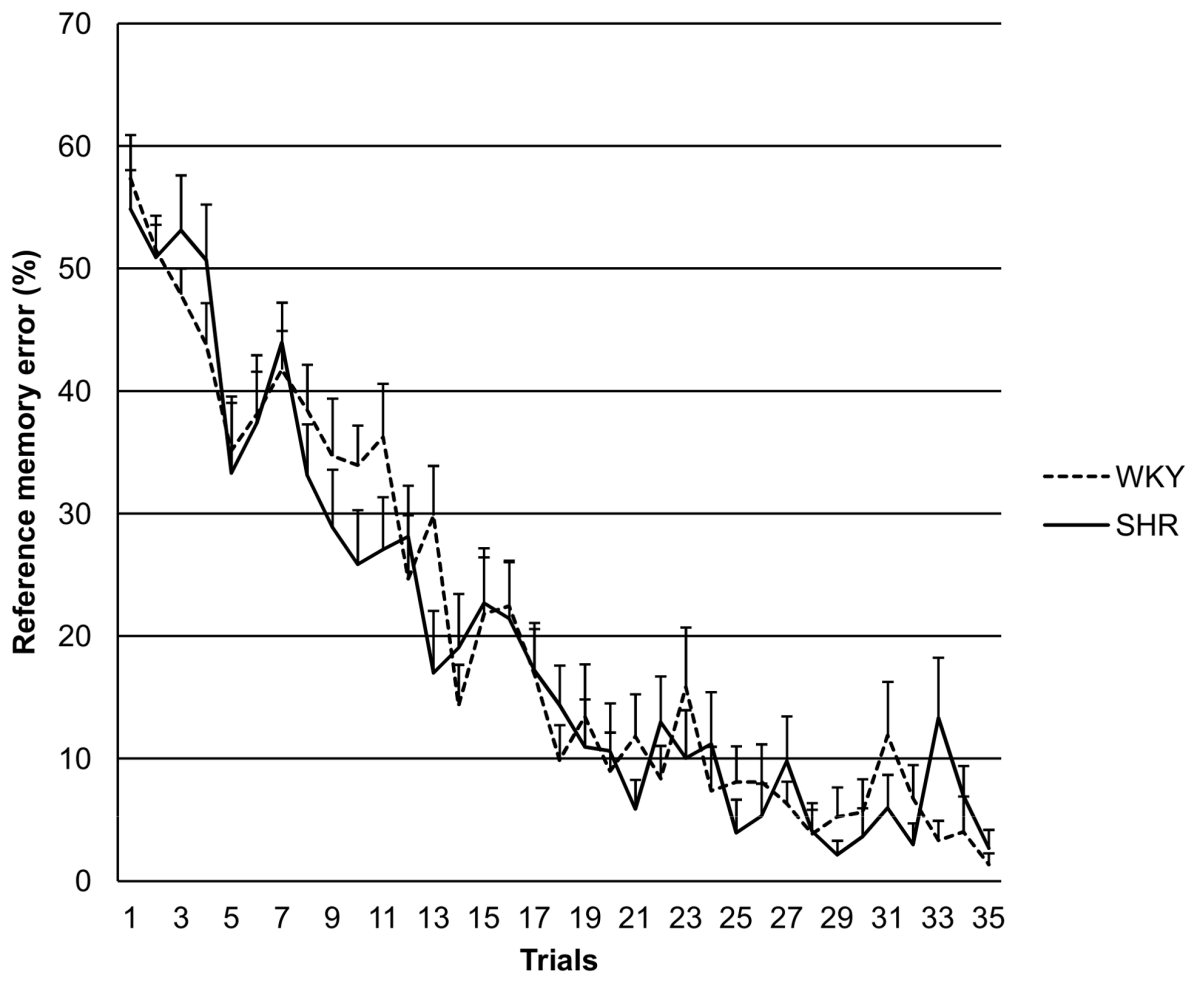
The reference memory error for WKY rats and SHRs. Values are means ± standard errors for each trial.

Figure 2 shows the route covered for the two groups in all 35 trials. The route covered was significantly different between SHRs and WKY rats (M ± SE: 232.8 ± 15.6 cm in SHRs; M ± SE: 174.9 ± 13.0 cm in WKY rats; *t*(28)=2.85, p=0.008) when the last 10 trials were analysed. Figure 3 presents the learning curve as indicated by reference memory error for all 35 trials. When using the last 10 trials ANOVA did not show any difference between the two groups in reference memory error or spatial working memory error (see Table 1). ANCOVA revealed a significant influence of the route covered on both the spatial working memory error (p=0.001) and the spatial reference memory error (p=0.001). There was a significant difference between groups in both spatial working memory error (p=0.001) and spatial reference memory error (p=0.018) when route covered was used as covariate (see Table 1). In addition, ANCOVA revealed that the covariate “route covered” explains 67.2% (Eta^2^ = 0.672) of the variance of the working memory error, while the group (Eta^2^ = 0.348) explains 34.8%. As for the reference memory error, 51% (Eta^2^ = 0.510) can be explained by the covariate “route covered” and 19% (Eta^2^ = 0.190) by the group.

**Table 1:** Effect of group on spatial working memory error and spatial reference memory error in WKY rats and SHRs (ANOVA); ANCOVA with route covered as a covariate.

	ANOVA			ANCOVA		
	M ± SE	F value	p value	adjusted M ± SE	F value	p value
Working memory error						
WKY (n=15)	6.34 ± 1.32			8.18 ± 0.69		
SHR (n=15)	6.07 ± 0.85	0.03	0.864	4.23 ± 0.69	14.38	0.001
Reference memory error						
WKY (n=15)	5.64 ± 1.38			7.45 ± 0.96		
SHR (n=15)	5.64 ± 1.11	0.00	0.998	3.84 ± 0.96	6.35	0.018

## Discussion

SHRs are known to show hyperactive behaviour [1,8,26,27] and alterations in spatial working and reference memory. For example, using a radial arm maze, Mook et al. [22] showed better working memory performance of SHRs compared to WKY rats, while Wyss et al. [16], Mori et al. [12] and Hernandez et al. [23] found a worse performance of SHRs. Using a water version of the radial arm maze, Clements and Wainwright [28] demonstrated spatial reference memory deficits in SHRs. However, when compared to the WKY rats, this spatial reference memory deficit was only seen at the end of the testing period. No difference in working memory error was observed by Clements and Wainwright [28]. These authors suggest that SHRs do not have a real spatial reference memory deficit and argue that the entries into wrong maze arms may be the result of hyperactive behaviour rather than cognitive deficits, i.e. the hyperactivity of SHRs may be a confounding factor when assessing cognitive performance. This is relevant for cognitive paradigms, such as the holeboard and water maze, with a pronounced locomotor component. Differences in test animals’ activity might explain the inconsistent results regarding spatial working and reference memory in previous studies. Locomotor activity needs therefore to be controlled for in future experiments.

In the present experiment, we found no difference in working memory error between the groups when the route covered was not used as a covariate. This finding is in accord with the study of Clements and Wainwright [28]. However, when using the route covered as a covariate we found a significantly lower working memory error in SHRs than in WKY rats which is in line with the study of Mook et al. [22]. ANCOVA showed a significant influence of the route covered on the working memory error. In addition, 67% of the variance of the working memory error can be explained by the route covered and only 34% by the group. In regard to the reference memory error we found no difference between the groups when the route covered was not taken into account. Using route covered as a covariate we found a lower reference memory error in the SHRs than in WKY rats. Approximately 51% of the variance of the reference memory error can be explained by the route covered and only 19% by the group. In summary, these results show that the variance of the cognitive parameters working memory error and reference memory error can be better explained by the covariate than by the factor group. Locomotor activity may therefore be a confounding factor when assessing cognitive performance in paradigms such as the holeboard, the water maze or radial arm maze. The present findings support the notion of Clements and Wainwright [28] who suggested that the cognitive deficits observed in SHRs are the result of their locomotor activity.

## Conclusion

In comparison to WKY rats, SHRs did not have any impairment in spatial working memory and reference memory. When the rats’ locomotor activity was taken into account, the SHRs’ working memory and reference memory were significantly better than in the control group. The rats’ activity appears to be a confounding factor at least in spatial memory tasks and should therefore be controlled for in future studies. Previous findings concerning spatial memory of SHRs should be interpreted with caution as they may have been confounded by an increase in locomotor activity. In the SHR model of ADHD, we were unable to demonstrate an impairment of working memory in a holeboard paradigm.
